# A Systematic Review of the Anxiolytic-Like Effects of Essential Oils in Animal Models

**DOI:** 10.3390/molecules201018620

**Published:** 2015-10-14

**Authors:** Damião Pergentino de Sousa, Palloma de Almeida Soares Hocayen, Luciana Nalone Andrade, Roberto Andreatini

**Affiliations:** 1 Departamento de Ciências Farmacêuticas, Universidade Federal da Paraíba, CP 5009, João Pessoa-PB, CEP 58051-970, Brazil; E-Mail: damiao_desousa@yahoo.com.br; 2 Departamento de Farmacologia, Universidade Federal do Paraná, CP 19031, Curitiba-PR, CEP 81540-970, Brazil; E-Mail: pallomaas@yahoo.com.br; 3 Departamento de Fisiologia, Universidade Federal de Sergipe, CEP 49100-000, São Cristóvão-SE, Brazil; E-Mail: lulisynalone@hotmail.com

**Keywords:** essential oil, terpene, natural products, anxiolytic, anxiety, tranquillizer, sedative, relaxant, elevated plus maze, lavender

## Abstract

The clinical efficacy of standardized essential oils (such as *Lavender officinalis*), in treating anxiety disorders strongly suggests that these natural products are an important candidate source for new anxiolytic drugs. A systematic review of essential oils, their bioactive constituents, and anxiolytic-like activity is conducted. The essential oil with the best profile is *Lavendula angustifolia*, which has already been tested in controlled clinical trials with positive results. *Citrus aurantium* using different routes of administration also showed significant effects in several animal models, and was corroborated by different research groups. Other promising essential oils are *Citrus sinensis* and bergamot oil, which showed certain clinical anxiolytic actions; along with *Achillea wilhemsii*, *Alpinia zerumbet*, *Citrus aurantium*, and *Spiranthera odoratissima*, which, like *Lavendula angustifolia*, appear to exert anxiolytic-like effects without GABA/benzodiazepine activity, thus differing in their mechanisms of action from the benzodiazepines. The anxiolytic activity of 25 compounds commonly found in essential oils is also discussed.

## 1. Introduction

Generalized anxiety disorder (GAD) is characterized by a high and free-floating anxiety, revealing both psychic (e.g., tension, insomnia, *etc.*), and somatic symptoms (e.g., muscle tension, dry mouth, *etc.*). GAD maintains a significant prevalence and causes important personal, family, and social impairments, which makes adequate treatment of GAD patients essential.

Pharmacological treatment of GAD usually employs benzodiazepines (e.g., diazepam and clonazepam), azaspirone (buspirone), and antidepressants (e.g., paroxetine). More recently, the anticonvulsant pregabalin has been introduced to treat GAD. However, all of these drug treatments have important drawbacks, such as abuse/dependence liability, retrograde amnesia (benzodiazepines), clinical effect delay (buspirone and antidepressants), sexual dysfunction (antidepressants), sedation (benzodiazepines and pregabalin), and dizziness (pregabalin), all of which affect clinical adherence [1]. Though it is not surprising that nearly 43% of patients with significant anxiety symptoms already use some form of alternative treatment [2], the above facts highlight the need for new anxiolytic drugs.

Medicinal plants are cited frequently as a potential source for new drugs, including for GAD. However, despite numerous pre-clinical studies, in well controlled studies very few medicinal plants have showed consistent clinical efficacy for GAD [3]. One interesting exception is a standardized essential oil from *Lavender officinalis*, called Silexan, which has been found effective for GAD patients [4], and reinforces the attention given to essential oils to source new GAD treatments. When we recognize that extensive pre-clinical research for discovery of new anxiolytic drugs has resulted disappointingly in very few molecules, their importance increases [5]. Certain essential oils have shown anxiolytic-like effects in animal models [6,7,8]. Essential oils are obtained from natural raw plant material thru steam distillation, from the epicarp of citrus fruits through mechanical processes, or by dry distillation after separation of the aqueous phase—if any—by physical processes (ISO 9235:2013). The pharmacological activity of essential oils on the central nervous system has been reported in previous reviews [9,10,11]. An earlier systematic review [6] focuses more on methodological issues. In the interim however, several studies have been publicized (Table 1). The aim of this review was to conduct a systematic investigation of pre-clinical essential oil studies in animal anxiety models.

## 2. Results and Discussion

The essential plant oils found with our search strategy where anxiolytic-like effect was found were: *Acantholippia deserticola*, *Achillea umbelata*, *Achillea wilhemsii*, *Alpinia zerumbet*, *Angelica sinensis*, *Chamaecypais obtuse*, *Casimiroa pringlei*, *Citrus aurantium*, *Citrus aurantium* subsp. bergomia (bergamot), *Citrus junos*, *Citrus latifolia*, *Citrus limon*, *Citrus reticulate*, *Citrus sinensis*, *Coriandrum sativum* var. microcarpum, *Cymbopogon citratus*, *Ducrosia anethifolia*, *Celastarus paniculatus*, *Chamaecyparis obtuse*, *Copaifera reticulata* Ducke, *Dennettia tripetala*, *Ducrosia anethifolia*, *Foeniculum vulgare*, *Lavendula angustifolia*, *Lippia alba*, *Ocimum sanctum* L., *Ocimum basilicum* L. essential oil, *Piper guineense*, *propolis*, *rose*, *Spiranthera odoratissima* A. St. Hil, *Santalum album* L., *Stachys tibetica*, and *Thujopsis dolabrata*. Table 1 shows the studies included, describing their main characteristics (essential oil, administration route, species and genus, animal model used, group control used, control for motor activity), and results. In addition, we present certain studies which evaluate the anxiolytic-like effects of constituents isolated from their essential oils (Table 2).

### 2.1. Abies sachalinensis

The effect of *Abies sachalinensis* essential oil (dark green needles) was evaluated in the elevated plus-maze [12]. Male mice treated acutely with inhaled *Abies sachalinensis* essential oil exhibited an increase in open arm exploration (% time spent and entries) in a 10-min session. However, when the essential oil was administered intraperitoneally it did not induce any change in the open arm explorations. These results indicate that the route of administration influences the anxiolytic-like effect of the essential oil, and the authors proposed that this difference may be related to the brain concentration of its constituents (e.g., α-pinene), and/or the olfactory sense [12].

### 2.2. Acantholippia deserticola

Female rats treated (i.p., acute, only during diestrous phase) with *Acantholippia deserticola* essential oil revealed increases in open arm exploration (% time and entries). However, these effects were seen at dose that also decreased square crossings in the open-field test, indicating motor impairment or sedative/depressant effect. Furthermore, the essential oil has a narrow effective range, with toxic effect (tonic-clonic convulsion) at a dose slightly above the effective dose in the elevated plus-maze [13].

### 2.3. Achillea umbelata

*Achillea umbelata* essential oil (aerial parts) was administered orally (acute) to male mice tested in the light/dark test [14]. An increase in time spent in the light side, and a decrease in the number of transitions was observed. The experimenters also described a certain paralyzing effect of the treatment. Moreover, the essential oil decreased motor activity in the open-field test. The authors suggested that the effect was more likely related to toxic effect than anxiolytic-like effect [14].

### 2.4. Achillea wilhemsii

Male rats treated acutely with *A. williemsii* essential oil (i.p.) displayed increased open arm exploration (number of entries, and % time spent) in the elevated plus-maze at a dose that did not change the total number of entries but decreased the closed arm entries [15]. The latter result indicates a motor impairment that may have influenced the anxiety indexes. Although flumazenil blocked the positive effect of diazepam (positive control), it did not change the effect of *A. williemsii*, indicating a non GABA/benzodiazepine mediation. Also, naloxone did not block the effect of *A. williemsii*.

**Table 1:** Summary of studies with essential oils in animal models of anxiety (only essential oils with at least one positive result are included).

Essential Oils	Administration	Specie	Anxiety Model	Observed Effect	Motor Activity	Mechanism of Action	Observation	Reference
*Abies sachalinensis*	Inhalation (acute) i.p. (acute)	Mouse	Elevated plus-maze	Anxiolytic-like No effect	Not evaluated		10-min session DR+	[12]
*Acantholippia deserticola*	i.p.	Rat	Elevated plus-maze	Anxiolytic-like?	Decrease		Female only Toxicity? DR+	[13]
*Achillea umbelata*	Oral (acute)	Mice	Light/Dark	Anxiolytic-like?	Decrease		Toxicity? DR+	[14]
*Achillea wilhemsii*	i.p. (acute)	Rat	Elevated plus-maze	Anxiolytic-like	Decrease?	Not mediated by BDZ Not mediated by opioid receptors	DR+	[15]
*Alpinia zerumbet*	i.p. (acute)	Mouse	Elevated plus-maze	No effect	Decrease		DR+	[16]
	Inhalation (acute)	Mouse	Elevated plus-maze	Anxiolytic-like	No change	Decrease 5-HTP and fluoxetine induced jumping (5-HT action)	Motor activity evaluated trough rearing DR+	[17]
	Inhalation (3 days) Inhalation (1 day)	Mouse	Elevated plus-maze Light/Dark	Anxiolytic-like No effect	No change No change		DR−	[18]
	Inhalation (5–150 min)	Mouse	Elevated plus-maze	Anxiolytic-like	No change		Anxiolytic-like effect is dependent of duration of inhalation (30–120 min) DR+	[12]
*Angelica sinensis*	Oral (acute)	Mouse	Elevated plus-maze Light/Dark Stress-induced hyperthermia	Anxiolytic-like Anxiolytic-like Anxiolytic-like	No change		Inverted U curve DR+	[19]
	Oral (acute)	Rat	Social Interaction	Putative Anxiolytic-like	Increase		Inverted U curve DR+	[19]
*Casimiroa pringlei*	Oral (acute)	Rat	Elevated plus-maze Holeboard	No effect (compared to control)	No change		Positive effect when compared to caffeine only DR+	[20]
*Celastarus paniculatus*	Oral (repeated)	Rat	Elevated plus-maze Vogel	Anxiolytic-like Anxiolytic-like	No change			[21]
*Chamaecyparis obtusa*	Inhaled (acute)	Mouse	Elevated plus-maze	Anxiolytic-like	Not evaluated		10-min session DR+	[22]
	Inhaled (repeated)	Rat	Elevated plus-maze	Reversed anxiogenic-like effect of mother separation	Not evaluated	Associated with IL-6 and Ccl2 cytokines reductions	DR−	[23]
*Citrus aurantium* L.	Oral	Mouse	Elevated plus-maze Open Field	Anxiolytic-like	No change		DR+	[24]
	Oral	Mouse	Marble-burying Light/Dark	Anxiolytic-like	No change		DR+	[25]
	Inhalation	rats	Social interaction Open Field Elevated plus-maze	Anxiolytic-like	No change		DR+	[26]
	Oral	Mouse	Light/Dark	Anxiolytic-like	No change	5-HT1A-receptors	DR+	[27]
	i.p. (acute)	Mouse	Elevated plus-maze	Anxiolytic-like	Not evaluated		DR+	[28]
	i.p. (acute)	Mouse	Elevated plus-maze	Anxiolytic-like	Not evaluated	GABA partial agonist?	Reduced diazepam anxiolytic-effect DR+	[29]
*Citrus aurantium* subsp. *bergamia*		Rat	Elevated plus-maze	Anxiolytic-like?	Increase?		inverted U-shaped curve DR+ decrease increase corticosterone induced by behavioral test	[30]
*Citrus junos*	Inhalation	Mouse	Elevated plus-maze Light/Dark	Anxiolytic-like Anxiolytic-like	No change		DR+	[31]
*Coriandrum sativum* var. *microcarpum*	Inhalation (repeated)	Rats	Elevated plus-maze	Decrease anxiogenic-like effect of icv beta-amyloid (1–42)	Not evaluated		Not tested in naive rats DR−	[32]
Lemon	Inhalation (continuous for 1 week)	Rat (male/female)	Elevated plus-maze	Anxiogenic-like	No change		DR−	[33]
	Inhalation	Mouse	Elevated plus-maze	Anxiolytic-like?	Decrease	5-HTergic (5-HT1A) GABA-A/BZP and DAminergic	DR−	[34]
*Citrus limon*	Oral (acute)	Mouse	Elevated plus-maze	Anxiolytic-like?	Decrease		inverted U-shaped curve DR+	[35]
*Citrus latifolia*	Oral	Mouse	Marble-burying Light/Dark	Anxiolytic-like Anxiolytic-like	No change		inverted U-shaped curve DR+	[36]
*Citrus reticulata*	Oral	Mouse	Marble-burying Light/Dark	Anxiolytic-like No effect	No change		DR+	[36]
*Citrus sinensis*	Inhalation	Rat	Light/Dark Elevated plus-maze	Anxiolytic-like Anxiolytic-like	No change		*Melaleuca alternifolia* essential oil used as neutral odor control DR+	[37]
*Copaifera reticulata*	Oral (acute)	Rat	Elevated plus-maze	Anxiolytic-like	No change		DR+	[38]
*Cymbopogon citratus*	Oral	Mouse	Open Field Elevated plus-maze Light/Dark	Anxiolytic-like	No change		DR+	[39]
	Oral	Mouse	Marble-burying Light/Dark	Anxiolytic-like	No change	GABA-A /BDZ	DR+	[40]
*Dennettia tripetala*	i.p. (acute)	Mice (male/Female)	Elevated plus-maze	Anxiolytic-like * (see observation)	Not evaluated		Inconsistency in data showed in figure and text Gender not considered DR+	[41]
*Ducrosia anethfolia*	Oral (acute)	Mouse	Elevated plus-maze	Anxiolytic-like	No change		DR+	[42]	
*Foeniculum vulgare*	Oral	Mouse	Elevated plus-maze Staircase test Open-field	Anxiolytic-like Anxiolytic-like Anxiolytic-like	No change		inverted U-shaped curve DR+	[43]
*Lavandula officinalis*	i.p.	Mouse	Geller conflict Vogel conflict	Anxiolytic-like Anxiolytic-like	No change		DR+	[44]
*Lavandula angustifolia*	Inhalation	Mouse	Elevated plus-maze	No effect			DR−	[34]
	Inhalation (24 h) Inhalation (7 days)	Gerbil (male/female)	Elevated plus-maze	Anxiolytic-like Anxiolytic-like			Anxiolytic in male and female Inclusion of ethological measures One-tailed test DR+	[45]
*Lavandula angustifolia*	Inhalation	rat	Open Field	Anxiolytic-like	Not evaluated (total locomotion)		Increase immobility (sedation) Inclusion one group also exposed during the open-field DR+	[46]
	Inhalation	rat	Open Field	Anxiolytic-like	No change		Reduction in c-fos increases with open field exposition DR−	[47]
	Inhalation	Sheep	Reaction to stress (isolation)	Mixed results	change		Nervous sheep: anxiogenic; calm sheep: anxiolytic-like effect DR−	[48]
	Inhalation	Mouse	Elevated plus-maze	Anxiolytic-like	No change		Anxiolytic-like effect correlated with linalool/linalyl acetate content DR−	[49]
	Inhalation	Mouse	Elevated plus-maze	Anxiolytic-like			Similar effect in stressed and non-stressed mice DR−	[50]
	Inhalation	Mouse	Elevated plus-maze Marble-burying	Anxiolytic-like Anxiolytic-like	No change	Serotonergic system (5-HT1A) Not mediated by GABA-A/BDZ	Neutral odor control DR+	[51]
	Inhalation	Mouse	Marble-burying	Anxiolytic-like	No change		Similar effects in anosmic and normal mice DR+	[52]
*Lavandula angustifolia*	Inhalation	Mouse	Elevated plus-maze	Anxiolytic-like		Increase hippocampal 5-HT turn-over	Similar effects in anosmic and normal mice DR−	[53]
*Lavandula angustifolia (silexan)*	i.p. (repeated)	rat	Elevated plus-maze Open Field Elevated zero-maze Social interaction test Novelty-induced suppressed feeding latency test	Anxiolytic-like Anxiolytic-like Anxiolytic-like Anxiolytic-like Anxiolytic-like	Mixed results		Some results could be influenced by motor activity changes DR+	[54]
	Oral (repeated)	Mouse	Elevated plus-maze	Anxiolytic-like	No change	Non selective inhibition of voltage operated calcium channels	DR+	[55]
*Lippia alba*	i.p.	Mouse	Elevated-plus-maze	Anxiolytic-like	No change		DR+	[56]
	i.p. (acute)	Rat	Elevated T-maze	Anxiolytic-like	No change		DR+	[57]
*Litsea cubeba*	Oral (7 days)	Mouse	Elevated plus-maze	Anxiolytic-like?	Decrease		DR+	[58]
*Ocimum gratissimum* L.	Inhalation (acute)	Mouse	Light/Dark	Anxiolytic-like?	Decrease		inverted U-shaped curve DR+	[59]
*Ocimun sanctum* L.	Inhalation (repeated)	Rat	elevated plus-maze	Decrease anxiogenic-like effect of icv beta-amyloid (1–42)	No change		Not tested in naive rats DR+	[60]
*Ocimum basilicum* L.	Inhalation (repeated)	Rat	elevated plus-maze	Decrease anxiogenic-like effect of icv beta-amyloid (1–42)	No change		Not tested in naive rats DR+	[60]
*Piper guineense*	Inhalation (acute)	Mouse	Light/Dark	Anxiolytic-like?	Mixed results		DR+	[61]
*Propolis*	Oral (14 days)	Mouse	Elevated plus-maze	Anxiolytic-like	No change		Effect in stressed mice DR+	[62]
		Rat	Elevated plus-maze Open-field	Anxiolytic-like?	Increase		Female (estrous phase not specified) DR+	[63]
*Rose centifolia*	i.p.	Mouse	Geller conflict Vogel	Anxiolytic-like Anxiolytic-like	No change		DR+	[64]
*Rose*	Inhaled	Rat	Elevated plus-maze	Anxiolytic-like	Not evaluated		DR+	[65]
	Inhalation (acute)	Mouse	Elevated plus-maze	No effect	No change		Ethanol as control DR−	[34]
*Rose damascena*	Prolonged Inhalation (24 h) Repeated Inhalation (14 days)	Gerbil	Elevated plus-maze Light/Dark	No effect Anxiolytic-like?	Mixed		DR−	[66]
*Santalum album* L.	Inhalation (acute)	Mouse	Elevated plus-maze	Anxiolytic-like in stressed mice	Not evaluated		No effect in non-stressed mice DR−	[67]
*Spiranthera odoratissima* A. St. Hil.	Oral (acute)	Mouse	Elevated plus-maze Light/Dark Open-field	Anxiolytic-like Anxiolytic-like Anxiolytic-like	No change	5-HTergic (5-HT1A) Not mediated by GABA-A/BDZ	DR+	[68]
*Stachys tibetica*	Oral (3 days) Oral (7 days)	Rat	Elevated plus-maze Social Interaction Light/Dark Holeboard Elevated plus-maze	Anxiolytic-like Anxiolytic-like Anxiolytic-like Anxiolytic-like Anxiolytic-like	No change		DR+	[69]
*Thujopsis dolabrata*			Elevated plus-maze	Anxiolytic-like	Decrease		10 min session DR+	[70]

Anxiolytic-like?—sedation/motor activity impairment could be a confounding variable; Toxicity?—Putative toxic effect; DR (dose/concentration-response design)—Plus (+) where at least two doses/concentrations tested; Minus (−) where only one dose/concentration tested; * Statistical significant difference cited in the text but not showed in the graphic.

**Table 2:** Constituents isolated from essential oils with anxiolytic-like effect.

Compound	Experimental Protocol	Anxiolytic-Like Effect and/or Mechanism	Animal Tested	Reference
*1,4-Cineole* 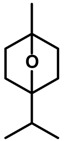	Elevated plus-maze test Holeboard test	Increased exploration of the open arms Increased head dipping	Mice	[71]
*α-Asarone* 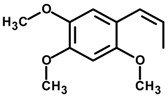	Elevated plus-maze test Holeboard test Open field test	Decreases in open-arm exploration Increased time spent for head dips Increase in the total number of line crossings	Rats	[72]
*α-Pinene* 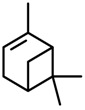	Elevated plus-maze test	Increased open arm exploration in the elevated plus maze	Mice	[73]
*β-Caryophyllene* 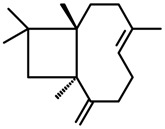	Elevated plus-maze test Light/dark test Marble-burying test Open-field test	Increased open arm exploration Increased time spent in light side, and number of transitions Decreased number of marbles buried Increased time spent in the center	Mice	[68,74]
*Citral* 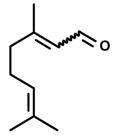	Elevated plus-maze test	No effect was observed	Mice	[75]
*Myrcene* 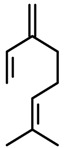				
*Carvacrol* 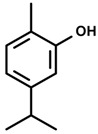	Elevated plus-maze test	Increased % time spent and % entries in the open arms	Mice	[76]
*Carvacryl acetate* 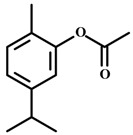	Elevated plus-maze test Light-dark box test Marble-burying test	Increased mouse motor activity Anxiolytic-like effect	Mice	[77]
(*R*)-(-)-*Carvone* 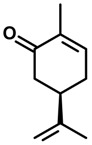	Elevated-T maze test	Reduced avoidance latency without any effect in escape time	Rats	[57]
*Citronellol* 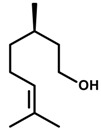	Geller conflict test	Increased punished behavior at dose that did not change unpunished behavior	Mice	[64]
*2-Phenethyl alcohol* 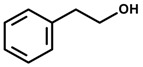	Vogel conflict tests	Increased punished licking		
*(E)-Methyl isoeugenol* 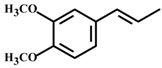	Elevated plus-maze test Light/dark test Open field test	Increased time spent and % entries in the open arms Increased number of transitions and time spent in light side Increased crossing of the center	Mice	[78]
*Nerol* 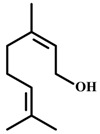	Elevated plus-maze test Open field test Light/dark test Rota rod test	Increased number of entries and time of permanence in the open arms Decrease in motor activity Increased time of permanence in the room clear No modification in time spent or number of falls in the revolving bar	Mice	[79]
*Isopropyl N-methylanthranilate* 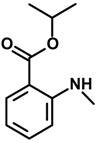	Light/dark test Open field test	Increased time spent in the light side without effect on the number of crossing	Mice	[80]
*Methyl N-methylanthranilate* 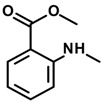				
*Isopulegol* 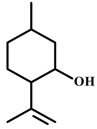	Elevated plus-maze test Holeboard test Open field test	Increased number of entries and time spent in the open arms Increase number of head dips Did not change the number of crossing	Mice	[81]
(*R*)-(+)-*Limonene* 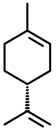	Elevated plus-maze test Light/dark test	Increase in time spent and in the number of entries in the open arms Increased time spent in the light side of the light/dark apparatus	Mice	[31,82]
(+)-*Limonene epoxide* 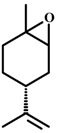	Marble burying test	Reduction in number of buried marbles	Mice	[83]
*Linalool* 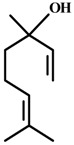	Light/dark test Elevated plus-maze test	Increased time spent in the light side and increased social interaction Increased number of visits to the open arms	Mice	[84]
*Linalool oxide* 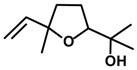	Light/dark test	Increased number of crossings and time spent in the light side	Mice	[85]
*Myrtenol* 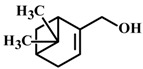	Elevated plus-maze test Light/dark test	Increase in open arm exploration Elevated time spent in the light side of light/dark apparatus	Rats	[86]
*Phytol* 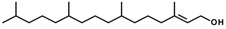	Elevated plus-maze test	Increased social interaction and decreased number of marbles buried	Mice	[87]
*Pulegone* 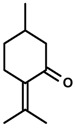	Elevated plus-maze test Open field test Rota rod test Grasping test Conditioning place preference (CPP) test	Increased mouse motor activity Increased ambulatory activity of mice in a dose-dependent and bell-shaped manner Decreased performance on the Rota rod apparatus Decreased grasping strength Decreased percentage of time spent in the least preferred compartment	Mice	[88]
*Thymol* 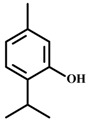	Brief mechanical restraint Open-field test	Reduced struggle latency and increased struggle bouts, which would be indicative of fear reduction No effect on motor activity	Quail	[89]
*Vanillin* 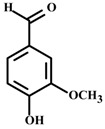	Elevated plus-maze test Bright and dark arena	Increase in the percentile ratio of open arm to total arm entries and reduction in the time spent in the closed arms Increased number of bright chamber entries, time spent, and rears in bright arena	Rats	[90]

### 2.5. Alpinia zerumbet

In the first study with *Alpinia zerumbet* essential oil (leaves), De Araújo and collaborators [16] observed no effect in mice (gender not cited) tested in the elevated plus-maze (50–100 mg/kg, i.p.), although they did find a sedative/depressant effect in the open-field test,. On the other hand, Murakami and collaborators [17] found that acute inhalation using *Alpinia zerumbet* essential oil (leaves) increased the time spent by male mice in the open arms of the elevated plus-maze at a dose which did not affect motor activity. The same group published two additional studies showing similar results. Satou and collaborators [18] observed that inhalation of this essential oil by mice increased the time spent in the open arms of the elevated plus-maze (three days of administration), this at a dose which did not change motor activity (calculated through rearing frequency in an observation box). Apparently, no significant effect was seen in the light/dark test (one day of administration). Satou and collaborators [91] also found that male mice treated with *Alpinia zerumbet* essential oil (leaves) at a single inhalation exhibit an increase in open arm exploration (% entries and time spent) in the elevated plus-maze, without changes in the total distance travelled. They also showed that this anxiolytic-like effect is dependent on inhalation time (90 to 120-min). Together, these results suggest an anxiolytic-like effect for *A. zerumbet* essential oil.

### 2.6. Angelica sinensis

Chen and collaborators [19] studied the effect of *A. sinensis* essential oil in mice tested in two animal models of anxiety (elevated plus-maze and light/dark transition), and a single test for detection of anxiolytic-like drugs (stress-induced hyperthermia). Mice treated orally with *A. sinensis* essential oil exhibited an increase in open arm exploration (% time spent in), and a decrease in protected head dips on the elevated plus-maze. An inverted U shaped response curve was observed. In the light/dark test, a higher dose of *A. sinensis* essential oil increased the number of transitions, while an intermediate dose increased the time spent in the lighted side. *A. sinensis* essential oil also reduced stress-induced hyperthermia, with a similar pattern being seen in the elevated-plus maze (U shaped curve). These effects were seen at doses that did not modify motor activity in the models (e.g., number of closed arm entries in the elevated plus-maze).

Min and collaborators [92] also evaluated the potential anxiolytic-like effect of *A. sinensis* essential oil in rats submitted to social interaction tests. *A. sinensis* (oral, acute) increased social interaction in unfamiliar and familiar test conditions, while diazepam was effective in unfamiliar test conditions only. Moreover, similar to what was observed by Chen and collaborators [19] in the elevated plus-maze, *A. sinensis* essential oil exhibited an inverted U shaped response in the social interaction test, since the high dose (42 mg/kg) only reduced aggressive behaviors, while the intermediate dose (21 mg/kg) increased social interaction. However, in the social interaction test, the increased motor activity induced by the intermediate and higher doses may be a confounding factor. In the holeboard test, only the lower dose increased head-dipping; an indication of anxiolytic-like effect.

### 2.7. Chamaecyparis obtusa

The putative anxiolytic-like effect of *Chamaecyparis obtusa* essential oil was tested in mother-separated male rats submitted to the elevated plus-maze test [23]. At 14 to 28 days (postnatal) the rats were separated from their mothers, and treated with essential oil (inhalation for 1 or 2 h, once a day). Fluoxetine was used as the control. The maternal separation induced an anxiogenic-like behavior in the elevated plus-maze (decreased percentage of time and entries in the open arms). Both *Chamaecypais obtuse* essential oil (1 h or 2 h), and fluoxetine reversed this anxiogenic-like effect. This anxiolytic-like effect was associated with reduction in interleukin 6 and Ccl2 cytokines. However, no control for motor activity change was used. Furthermore, the authors used a modified criteria for arm entry in the elevated plus-maze (2 paws instead the common 4 paws criteria).

Kasuya and collaborators [22] evaluated the effect of *C. obtusa* essential oil (inhalation for 90 min) in isolated male mice submitted to elevated plus-maze. They observed an increase in open arm exploration (% time and entries) in a 10-min session, associated with an increase in fast nerve growth factor receptor (NGFR). This effect in the elevated plus-maze was dose dependent with an inverted U shaped curve. Although these results could suggest an anxiolytic-like effect, no measurement of motor activity was described.

### 2.8. Casimiroa pringlei

Oral acute administration of *Casimiroa pringlei* essential oil in male rats induced an increase in % time spent in the open arms (elevated plus-maze), and increased holes exploration in the holeboard test, the dosage did not impair motor activity in the open-field [20]. However, these effects were significantly different from rats treated with caffeine alone, which did not permit conclusions with regard to its effect on anxiety.

### 2.9. Celastarus paniculatus

Rajkumar and collaborators [21] observed that rats (male and female) treated for 14 days (p.o.) with oil from *Celastarus paniculatus* seeds showed an increase in punished licking (Vogel’s test) and open arms exploration in the elevated plus-maze, without effect on motor activity in the open-field test. These results indicated an anxiolytic-like effect for this essential oil.

### 2.10. Citrus Genus

#### 2.10.1. *Citrus* sp.

Umezu [93] studied the effect of acute administration (i.p.) of several essential oils in mice submitted to conflict procedures (Geller and Vogel tests), revealing no effect for either orange oil (*Citrus* sp.), chamomile (*Matricaria chamomilla*), or Ylang-ylang (*Cananga odorata*). The positive effect observed with diazepam and rose oil (see below) indicated the procedure’s sensitivity.

#### 2.10.2. *Citrus aurantium* (Sour/Bitter Orange)

Carvalho-Freitas and Costa [24] observed that *C. aurantium* essential oil administration (acute oral) in mice increased exploration of the open arms (time spent) in the elevated plus-maze at a dose that did not impair motor activity (open-field and rota rod test), which is indicative of anxiolytic-like effect.

Pultrini and collaborators [25] studied the effect of *C. aurantium* in acute and repeated (15 days) oral administration in mice. The acute administrations induced an anxiolytic-like effect in the light/dark transition tests (increased time spent in the light side, and in the number of transitions) and in marble burying (decreased number of marbles buried), while repeated administration showed effects in the marble-burying test only. Repeated diazepam administrations did not increase light/dark transitions. For the rota rod tests, no *C. aurantium* effect was seen, suggesting no motor impairment. Thus, the results suggest an anxiolytic-like effect for acute and repeated *C. aurantium* essential oil treatment.

Costa and collaborators [27] also observed that both acute and repeated (14 day, p.o.) of *C. aurantium* essential oil administration induced anxiolytic-like effect in the light/dark transition model. Acute and chronic administrations increased both time spent in the light side, and the number of transitions, at a dosage that did not impair the motor activity of the mice [27]. Both in acute and chronic treatments, the lower doses were effective while higher doses were not. This anxiolytic-like effect of *C. aurantium* essential oil was reversed by 5-HT1A antagonist WAY100635 but not by flumazenil, a benzodiazepine antagonist, suggesting serotonergic mediation. Moreover, no effect on dopamine and/or serotonin levels was seen in the cortex, hypothalamus, pons, or striatum.

In this line, inhaled *C. aurantium* essential oil increased social interactions for rats (time spent in active social interaction), and increased exploration time in the open arms of the elevated plus-maze, suggesting an anxiolytic-like effect at a dose that did not impair motor activity in the open-field test [26].

Saketi and collaborators [28] observed that acute administration (i.p.) of *C. aurantium* L. essential oil in male mice increased open arm explorations (percentage of time spent and percentage of entries) in the elevated plus-maze. Fluoxetine exhibited the same anxiolytic effects. The authors claim that the effect of *C. aurantium* L. is related to serotonergic transmission based on a fluoxetine + *C. aurantium* L. interaction. However, *C. aurantium* L. did not change the anxiolytic effect of fluoxetine in the elevated plus-maze, suggesting no drug interaction. Moreover, no measurement of motor activity was presented in the results.

The same group evaluated the co-administration effects of *C. aurantium* L. with diazepam [29]. They found that *C. aurantium* L. essential oil (i.p.) increased the open arms exploration (increase of percentage time spent) of male mice submitted to the elevated plus-maze. Although diazepam increased open arms exploration (percentage of time spent and percentage of entries), co-administration with *C. aurantium* L. reduced the anxiolytic effect of diazepam. These results indicate that *C. aurantium* L. may exert an anxiolytic-like effect acting as partial agonist at the GABA-A receptor/benzodiazepine site. However, flumazenil did not alter the effect of *C. aurantium* [27]. One observation concerning this study is that the authors cited the total number of entries (closed + open) as being used for motor activity measurement, but there is no further mention of it in the text.

#### 2.10.3. *Citrus aurantium* subsp. *bergamia* (Bergamot)

Saiyudthong and Marsdem [30] evaluated the effect of bergamot oil in the elevated plus-maze and holeboard tests. Male rats treated with bergamot oil (inhalation for 7 min) showed increase in open arm exploration (% time and % entries) in the elevated plus-maze, and increased head dipping in the holeboard test. The dose-response curve exhibited an inverted U shape in both models, with the intermediate dose showing better results. Further, the higher dose increased the total number of arm entries, which might indicate an excitatory effect, although this parameter may be influenced by anxiety changes. No other measurement of motor activity was taken. It is interesting that bergamot oil and diazepam also decreased corticosterone levels after the elevated plus-maze test. Thus, bergamot essential oil exhibits an anxiolytic-like profile, although future studies must evaluate motor activity properly. This putative anxiolytic-like effect was corroborated in a clinical trial showing that bergamot oil inhalation reduces anxiety in patients awaiting minor surgery [94]. Interestingly, an *in vivo* study using micro-dialysis, observed that systemic bergamot oil administration (i.p.) increased aspartate, taurine, and glycine release in the hippocampus. However, local bergamot administration also increased glutamate and hippocampal GABA release [95].

#### 2.10.4. *Citrus latifolia* and *C. reticulata*

Gargano and collaborators [36] studied the effect of systemic (oral) administrations of *C. latifolia* (containing 58% limonene and 13% beta-pinene), and *C. reticulata* (containing 90% limonene) essential oils in the marble-burying test and the light/dark test. It was observed that mice treated with *C. latifolia* decreased the number of marbles buried (marble burying test) and increased the time spent in the light side (light/dark test) with an inverted U-shaped curve. The dosage did not impair performance in the rota-rod test. *C. reticulata* was effective only in the marble burying test (reduced number of marbles buried). Imipramine (marble burying) and diazepam (light/dark test) were used as positive controls. The data suggest an anxiolytic-like effect for both essential oils, but with different profiles (perhaps reflecting differing mechanisms of action).

#### 2.10.5. *Citrus junos*

Satou and collaborators [31] evaluated the effect of *C. junos* essential oil from pericarps in isolated male mice tested in the light/dark test and the elevated plus-maze. Apparently the same mice were tested in the light/dark test (first day), open-field (second day) and elevated plus-maze (third day). Inhaled *C. junos* essential oil increased the time spent and the number of entries in the light side of the light/dark apparatus. In the elevated plus-maze, the essential oil induced an increase in open arms exploration (time spent and % entries). These effects were seen at doses that did not impair open-field activity, indicating an anxiolytic-like effect for *C. junos* essential oil.

#### 2.10.6. *Citrus limon*

Inhaled lemon oil increased the time spent and the entries on the open arms of mice tested on the elevated plus-maze, without effects on motor activity (number of entries in closed arms), suggesting an anxiolytic-like effect [34]. However, in the same study, lemon oil inhalation reduced locomotion and exploration in the open field, which would be suggestive of a sedative/depressant effect. Considering that flumazenil (an antagonist of the benzodiazepine site at the GABA-A receptor), WAY 100635 (a 5-HT 1A receptor antagonist), and apomorphine (a non-selective dopamine agonist) all blocked lemon oil’s effect in the elevated plus-maze, the effect may be meditated by either GABA-A/benzodiazepine, serotonergic, and/or dopaminergic neurotransmissions.

On the other hand, Ceccarelli and collaborators [33] evaluated the effects of prolonged lemon essential oil exposition in male and female rats. The rats were exposed throughout one week (continuous home-cage exposure), and then tested in the elevated plus-maze. Both male and female rats exhibited a reduction in open arms exploration (% time spent in the open arms), indicating an anxiogenic-like effect. It has been previously shown that repeated stressful stimuli have anxiogenic-like effects [96], and thus, continuous lemon essential oil exposition may be a stressful stimulus inducing anxiogenic-like effect.

Using *Citrus limon* essential oil (from the leaves), containing limonene (52.7%) and linalool (1.7%), Lopes Campelo and collaborators [35] observed mixed results in the elevated plus-maze. At a low dose (50 mg/kg, p.o.) mice increased % time spent in the open arm, but decreased the percentage of open arm entries, while an intermediate dose (100 mg/kg) increased both parameters, and the highest dose (150 mg/kg) decreased both parameters. However, all doses decreased motor activity in the open field, suggesting a sedative/depressant effect that could interfere with the elevated plus-maze behavior.

#### 2.10.7. *Citrus sinensis* (Sweet Orange)

Inhalation of *Citrus sinensis* essential oil (sweet orange, containing 97% limonene) by male rats induced an increase in open arm exploration (% time spent and % number of entries) in the elevated plus-maze, and the % time spent in the lit chamber of the light/dark test [37]. There was no effect on motor activity, as measured by the total distance travelled in the elevated plus-maze. *Melaleuca alternifolia* essential oil was used as a neutral odor control. These results suggest an anxiolytic-like effect, which was corroborated in a clinical study with normal volunteers submitted to an anxiety-provoking experimental situation [97].

### 2.11. Copaifera reticulata Ducke

It was found that acute administration (p.o.) of *Copaifera reticulata* Ducke essential oil in rats increased conventional parameters of open arm exploration (% time spent and % entries), and ethological measures (rearing, peeping out, and end open arm activity) in the elevated plus-maze. No change was seen in the number of closed arm entries, indicating no impairment of motor activity [38]. These results suggest an anxiolytic-like effect for this oil.

### 2.12. Coriandrum sativum Var. microcarpum

Volatile oil extracted from *Coriandrum sativum* var. *microcarpum* was tested for anxiolytic-like action in an animal model of Alzheimer disease [32]. This oil contains linalool (69%) and α-pinene (6.5%). Male rats treated with β-amyloid (1–42) were submitted to an elevated plus-maze 60 min after the last volatile coriander oil inhalation (21 days, 60 min each day). It was observed that β-amyloid (1–42) decreased open arms exploration (percentage of time spent and number of open arm entries), and that volatile coriander oil reversed these changes. Although these results may suggest an anxiolytic-like effect, the influence of a putative motor impairment must be evaluated. Moreover, there was no group treated exclusively with volatile coriander oil.

### 2.13. Cymbopogon citratus

Male mice treated orally with *C. citratus* essential oil (leaves) (mainly composed of citral and β-myrcene) displayed increased open arm exploration (% entries and time spent in the open arms) in the elevated plus-maze, and time spent in the light side of the light/dark test [39]. No motor effect was seen in the open-field or rota-rod test. The pattern was similar to the positive control diazepam. Thus, the results suggested an anxiolytic-like effect for this essential oil. Costa and collaborators [40] also observed an anxiolytic-like effect in the light/dark test (increased time spent in the lighted side, and number of transitions at a dosage which did not impair rota rod performance), after acute administration. This effect was not seen after repeated (21 days) treatment, suggesting tolerance development. Furthermore, the anxiolytic-like effect appeared to be mediated by GABA-A/benzodiazepine transmission, since it was blocked by flumazenil but not by WAY100635 pretreatment. However, it is interesting to note that Vale and collaborators [75] did not observe anxiolytic-like effect in the elevated plus-maze with isolated citral, limonene, or myrcene, which suggests that other constituents contribute to the anxiolytic-like effect of *C. citratus*. In this study, diazepam, used as a positive control, increased open arms exploration, which corroborate the sensitivity of the procedure used.

### 2.14. Dennettia tripetala

Oyemitan and collaborators [41] studied the effect of *D. tripetala* essential oil and its main compound 1-nitro-2-phenylethane (BPNE) in the elevated plus-maze. Male and female mice treated with *D. tripetala* essential oil and BPNE (both i.p.) apparently showed an increase in open arm exploration (% arm entries and % time spent). These results suggest that the anxiolytic-like effect of *D. tripetala* essential oil is related to BPNE. However, no measure of motor activity was taken and some problems in the manuscript (e.g., significant effects of the essential oil described in the text were not shown in the figure concerning the elevated plus-maze test) making it difficult to draw an overall conclusion.

### 2.15. Ducrosia anethifolia

Evaluating the potential anxiolytic-like effect of *Ducrosis anethifolia*, essential oil oral administration in male mice, Hajhashemi and collaborators [42] found an increase in open arm exploration (time spent and entries) in the elevated plus-maze, at a dosage that did not impair motor activity (number of beam breaks in the automated chamber). This profile suggests an anxiolytic-like effect for *Ducrosis anethifolia* essential oil.

### 2.16. Foeniculum vulgare

Mesfin and collaborators [43] studied the putative anxiolytic-like effect of essential oil from aerial parts of *Foeniculum vulgare* in oral administrations to male mice. Acute *Foeniculum vulgare* essential oil administrations increased open arms exploration (% time spent and entries) in the elevated plus-maze, and decreased rearing in the staircase test. Furthermore, the essential oil increased center exploration (number of square crossings and time spent) in the open-field test. These effects showed an inverted U shape dose-response curve, and they were seen at doses that did not change motor activity in the elevated plus-maze (number of closed arm entries), staircase test (number of steps climbed), or open-field test (total number of square crossings). Thus, the results indicated an anxiolytic-like effect for *Foeniculum vulgare* essential oil [43]. This essential oil presents among its constituents certain substances that have also displayed anxiolytic-like effect such as: limonene, α-pinene, 1,8-cineole, and, at a lower concentration, linalool (see below).

### 2.17. Lavendula angustifolia

Umezu and collaborators [44] evaluated the effect of systemic lavender oil administration (i.p.) to mice in two conflict tests: the Geller and Vogel conflict tests. They observed that lavender had an anti-conflict effect: increased punished response at a dose that did not change unpunished response in the Geller test, and increased punished licking in the Vogel test. Further, the effects of the main constituents of lavender essential oil (linalool, linalyl acetate, borneol, camphene, cineol, terpinen-4-ol, α-pinene and β-myrcene) were also evaluated, and the only compound that showed a clearly anti-conflict effect was linalool. These results suggest that linalool may be the main active component of lavender.

Bradley and collaborators [45] studied the effect of lavender in Mongolian gerbils submitted to an adapted version of the elevated plus-maze. The use of gerbils is based on findings that this specie has a greater homology to humans in respect to the distribution of neurokinin-1 (NK-1) receptors than rats and mice. Male and female Mongolian gerbils were bred in an enriched environment with food supplemented by fruit and sunflower seeds; the gerbils were exposed to inhaled lavender continuously for 24 h, or for 7 days. Although some slight differences were seen between male and female gerbils, on the whole, acute lavender treatment exerted anxiolytic-like effects in ethological measures (e.g., decrease risk assessment behavior), however not in spatiotemporal measures (% entries or % time spent in open arms). On the other hand, chronic continuous lavender exposure increased the % entries in the open arms, and decreased the stretch-attend posture (another ethological measure). These results suggest that lavender’s anxiolytic-like effect did not decrease with repeated exposure, but appears to increase. It must be noted that the data were analyzed statistically using a one-tailed test, which may have influenced the conclusions.

Shaw and collaborators [46] suggested an anxiolytic-like effect for lavender essential oil using the open-field test. Male rats were exposed to various schedules of lavender essential oil inhalation and at 30 min or 1 h, before or during the test; all rats received an injection previous to the inhalation procedure. The higher doses of lavender reduced peripheral movement and defecation, effects that were also seen in the diazepam treated group. However, no data was given for central (considered the measure related to anxiety), or total motor activity (indicative of sedative/depressant effect and/or motor impairment). Moreover, certain lavender groups displayed decreased mobility, indicating for the oil a sedative or depressant, rather than an anxiolytic-like effect. This interpretation is consistent with reductions in rearing and grooming as seen in some of the experiments in this study.

Another study from Shaw and collaborators [47] evaluated the effect of lavender essential oil and chlordiazepoxide in c-fos expression after open field exposition testing. Again, lavender essential oil (inhaled for 1 h), and chlordiazepoxide reduced peripheral movement and defecation of the rats. However, lavender in this study did not affect mobility. Further, lavender and chlordiazepoxide decreased open-field induced increased c-fos expression in the paraventricular nucleus of hypothalamus, and in the dorsomedial hypothalamic nucleus. In addition, chlordiazepoxide also decreased open-field induced increased c-fos expression in the ventromedial hypothalamus, lateral hypothalamic area, accumbens shell and core, and the caudate-putamen striatum. The authors concluded that lavender exert anxiolytic-like effects which may be mediated by a different neural substrate than benzodiazepines.

Chioca and collaborators [51] observed that male mice acutely exposed to inhaled lavender essential oil displayed reduced marble-burying behavior and increased open arm exploration (% number of entries and time spent in the open arms), an effect that was not seen with amyl acetate (banana odor), that is used as a negative odor control (odor not inducing behavior changes). Diazepam, used as positive control, exerted anxiolytic effect in both models. The effect of lavender on marble-burying was not blocked by picrotoxin (GABA antagonist) pre-treatment, nor changed [^3^H] flunitrazepam binding. On the other hand, WAY 100635 (a 5-HT1A antagonist) blocked the anxiolytic-like effect of lavender in marble-burying test. Lavender essential oil also reduced 8-OH-DPAT (5-HT1A agonist) induced serotonergic syndrome. These results indicate that the anxiolytic-like effect of lavender essential oil may be mediated by serotonergic transmission.

A subsequent study by the same group replicated the anxiolytic-like effect of lavender essential oil in the marble-burying test, and it was observed that this effect was similar in mice with normal olfactory function, as compared to mice with anosmia, which suggests that odor perception was not crucial. The normal (non-anosmic), and anosmic mice (both treated with vehicle) results did not differ [52]. Using a similar approach, Takahashi and collaborators [53] observed that anosmic and non-anosmic male mice showed similar behavior in the elevated plus-maze after lavender inhalation. However, anosmic mice showed increased open arms exploration (time spent) in the elevated plus-maze; beyond that of non-anosmic mice. Inhaled lavender essential oil induced an anxiolytic-like effect in normal mice compared to vehicle treated mice. They also found an increase in hippocampal serotonin turn-over after lavender inhalation, reinforcing the proposal of a serotonergic mediation of the anxiolytic-like effects of lavender [51].

In a very interesting study, Takahsahi and collaborators [49] evaluated the anxiolytic-like effect of essential oils from different species of lavender (e.g., *L. officinallis* and *L. latifolia*), which differ in linalool and linalyl acetate contents. They observed that male mice treated with inhaled lavender essential oils that contain linalool and linalyl acetate spent more time in the open arms of the elevated plus-maze than the controls. These effects were seen without changes in the distance travelled in the maze, indicating an anxiolytic-like effect. However, there is no relationship between linalool content and anxiolytic-like effect, yet a positive correlation (r = +82) was seen between linalool and linalyl acetate content and increased open arm exploration. These results suggest that linalyl acetate, which showed no anxiolytic-like action *per se*, increased the anxiolytic-like effect of linalool.

Another study from Takahashi and co-workers [50] found that lavender essential oil inhalation exerted anxiolytic-like effect in the elevated plus-maze (10-min session), both in non-stressed and stressed male mice. However, stress (water immersion for 24 h, 1–2 cm deep) had no effect on elevated plus-maze behaviors. The divergent effects of lavender in stressed and non-stressed mice are observed in the expression of mRNA of some proteins (fast nerve growth factor receptor, NGFR, and the activity regulated cytoskeletal-associated protein, Arc).

Silexan is a standardized essential oil produced from *L. angustifolia* flowers containing 36% linalool and 34% linalyl acetate [55]. Repeated administration (7 days, i.p.) of Silexan showed anxiolytic-like effects in male rats in the elevated plus-maze (increase open arms exploration), zero-maze (increased exploration of open areas), social interaction (increased social interaction in a familiar environment), novelty-induced suppression feeding (decreased latency for eating starts in a novel environment), and the open-field square (increased central area exploration). However, some contradictory effects on motor activity were seen: increased square crossing in the open-field test (excitatory effect), decreased number of closed arm entries in the elevated plus-maze (motor impairment), and no effect on the square closed-field arena with light beam sensors [54]. Overall, these results suggest an anxiolytic-like effect, although it might be influenced by motor activity changes.

An anxiolytic-like effect for Silexan was also found by Schuwald and collaborators [55]. The authors observed that male mice treated with Silexan (orally for 3 days) showed an increase in open arms exploration (time spent and entries in open arms) in the elevated plus-maze. Diazepam and pregabalin were used as positive controls and exhibited the same effects. They also observed that the anxiolytic-like effect of Silexan is related to non-specific inhibition of voltage operated calcium channels showing similarities with pregabalin (although Silexan did not bind to the α2-δ subunits of the P/Q-type voltage operated calcium channels, the target of pregabalin). Finally, the authors stated that Silexan did not bind to SERT, DAT, NET, MAO-A, or GABA-A receptors. Thus, action on calcium homeostasis may contribute to the anxiolytic-like effect of Silexan (and thus, lavender essential oil).

It is interesting to note that rats trained to discriminate between saline and diazepam (two-lever operant behavior) did not generalize to the diazepam cue when treated with Silexan (3–30 mg/kg, i.p.), suggesting a that Silexan induces a different interoceptive stimuli [98]. These results reinforce the hypothesis that Silexan (thus, lavender essential oil) did not share the same mechanism of action as benzodiazepines [47,51,55].

Certain negative results were also found. Inhalation of lavender oil did not increase open arm explorations of mice tested on the elevated plus-maze [34]. Hawken and collaborators [48] observed that lavender essential oil exerted opposite effects in sheep for their stress response: lavender increases agitation, vocalization, and escape attempts in isolated stressed “nervous sheep” (sheep that exhibit increase motor activity and vocalization frequency when isolated or in human presence). However, lavender essential oil decreases these behaviors in “calm sheep”. “Nervous sheep” that inhaled lavender also showed increased plasma cortisol at 30 min after isolation stress. These unexpected findings in “nervous sheep” could be related to systemic delivery of the essential oil (individual mask), or to odor novelty, although plasma cortisol levels (before isolation testing, and after lavender administration) did not differ from the control. The authors proposed that the divergent response to stress (isolation and human proximity) is genetically determined; therefore genetic background may also influence the response to lavender essential oil. Moreover, considering that only a single concentration of lavender essential oil was administered, it may have been too low to reduce the stress reaction in “nervous sheep”. Future replications of this interesting bi-directional effect of lavender essential oil in a standardized rodent animal model for anxiety, while using a neutral odor and dose/concentration-response design would be very important.

Silexan has shown clinical anxiolytic effect in controlled studies with GAD patients [99,100], and those with sub-syndrome anxiety disorder [4]. Further, Silexan administration shows reduced 5-HT1A receptor binding in certain brain areas (e.g., hippocampus) in healthy volunteers [101], reinforcing the hypothesis that serotonin mediates the anxiolytic effect of lavender essential oil.

### 2.18. Lippia alba

Hatano and collaborators [57] administered *Lippia alba* essential oil (i.p.) to male rats which were then submitted to the elevated-T maze. *L. alba* reduced avoidance latency without any effect on escape time, a profile similar to the reference drug diazepam. Moreover, *L. alba* essential oil did not impair motor activity in the open-field test. Vale and collaborators [56] observed that acute administration of three chemotypes of *Lippia alba* essential oil (i.p.) increased male mice exploration of open arms (% entries and % time spent) in the elevated plus-maze. An anesthetic effect study of *Lippia alba* essential oil on silver catfish (*Rhamdia quelen*), suggested a GABAergic effect [102], explaining its anxiolytic-like effect. Taken together, the results suggest an anxiolytic-like effect for *L. alba* essential oil.

### 2.19. Litsea cubeba

Male mice treated orally for seven days with *Litsea cubeba* essential oil showed increased open arms exploration (time spent and number of entries), and a decrease in total distance traveled in the open arms of the elevated plus-maze test [58]. This result suggests a sedative effect influencing the behavior of mice in the elevated plus-maze.

### 2.20. Ocimum basilicum L. and Ocimun sanctum L. Essential Oil

Gradinariu and collaborators [60] evaluated the effects of *O. sanctum* L and *O. basilicum* L essential oils in male rats treated (i.c.v.) with beta-amyloid (1–42). Similar to that observed by Cioanca and collaborators [32], beta-amyloid (1–42) induced an anxiogenic-like behavior in the elevated plus-maze (decreasing the number of entries in the open arms). *O. sanctum* L. and *O. basilicum* L. (essential oil inhalations, 60 min per day, for 21 days) reversed this effect at doses that did not change motor activity (number of crossing in the elevated plus-maze), while increasing the percentage of open arms time spent. These essential oils contain linalool, and 1,8-cineole, which may well contribute to the behavioral effects [60]. However, there was no group treated only with essential oils.

### 2.21. Ocimum gratissimum L.

Tankam and Ito [59] administered the essential oil from *O. gratissimun* L to male mice tested in the light/dark and open-field tests. The essential oil was administered by inhalation during the behavioral test (embedded filter paper disk adhered to apparatus wall). It was observed that treated mice showed an increase in the number of transitions and time spent in the light side of the light/dark apparatus. The dose-response curve showed an inverted U shape. However, *O. gratissimun* essential oil administration also decreased motor activity in the open-field test, indicating a sedative/depressant effect that could contribute to increased time in the light side of the light/dark apparatus, but would impair the number of transitions (the opposite of the result observed). It is interesting to note that thymol, the main component of *O. gratissimun* essential oil, also showed anxiolytic-like effects (see below).

### 2.22. Piper guineense

Inhaled essential oil from dried fruits of *P. guineense* (containing linalool and 3,5-dimethoxytoluene) increased the time spent by mice in the light side of the light/dark apparatus and the number of transitions [61]. However, mixed results were seen for motor activity, since the essential oil reduced the number of crossing in the open-field test (indicating impairment) although not changing latency to the first transition in the light/dark test (indicating absence of impairment).

### 2.23. Propolis

Li and collaborators [62] studied the effect of propolis essential oil on stressed male mice (restraint stress) submitted to the elevated plus-maze. Propolis (administered orally for 14 days) reversed the anxiogenic-like effects of restraint stress in the elevated plus-maze test (decreased % time and entries spent in the open arms, and any increases in protective head dipping and rearing). These effects were seen at doses that did not change motor activity in the open-field testing (number of transitions), or in the elevated plus-maze test (number of closed arms entries). Propolis also reversed increases in plasma corticosterone and ACTH when induced by restraint stress.

Another study also showed an anxiolytic-like effect for propolis essential oil. Reis and collaborators [63] observed that female rats (estrous phase not specified) treated (intraperitoneally) with propolis increased center exploration in the open-field, and open arm exploration (% time and entries) in the elevated plus-maze tests. However, sometimes propolis also increased motor activity (distance travelled in the open-field test and number of closed arm entries in the elevated plus-maze) which may have influenced the results. Thus, propolis may exert anxiolytic-like effects, although motor activity changes must be observed to draw a clearer conclusion.

### 2.24. Rose

Umezu and collaborators [64] evaluated the effect of rose oil (*Rose centifolia*) and diazepam (both acute, i.p.) in the Geller conflict and Vogel conflict tests. Both treatments increased the punished behavior of mice in both tests, and were without effect for unpunished behavior in the Geller test, suggesting an anxiolytic-like effect for rose oil. Inhaled rose oil, (acute, 7 min), caused an increase in exploration of the open arms (time spent and number of entries) in the elevated plus-maze test although no measurement of motor activity was taken [65].

Bradley and collaborators [66] evaluated the behavioral effects on gerbils (male and female) of prolonged single (24 h), and repeated (2 weeks) rose oil (*Rose damascena*) inhalation. They did not observe any significant effect on % time and entries into the open arms of the elevated plus-maze, but there were effects on ethological measures (increases in head dips and rearing). On the other hand, they observed an increase in % time spent in the light side of the light/dark test (no male/female difference was found). However, rose oil also increased latency to first entrance in the dark compartment, which has been related to a sedative/depressant effect. Diazepam was effective in both models, corroborating the sensitivity of the procedure and suggesting that different mechanisms of action mediate the effects of benzodiazepine, *vs.* the effects of rose oil.

Contrary to studies showing anxiolytic-like effects for rose oil, Komiya and collaborators [34] observed that inhaled rose oil did not increase the time spent or the number of entries of mice in the open arm of the elevated plus-maze test. Yet, *C. limon* increased open-arm exploration, which confirmed the sensitivity of the procedure.

Thus, the results with rose oil are mixed and they may be influenced by a sedative/depressant effect. However, some of its main constituents (citronellol and 2-phenethyl alcohol) have shown anxiolytic-like effect.

### 2.25. Santalum album L.

Sandalwood oil (*Santalum album* L.) was studied in stressed male mice submitted to the elevated plus-maze [67]. Stressed mice were isolated for 1 week and immersed in 1-cm deep water for 24h. Stressed mice treated with *Santalum album* L oil (90-min inhalation) showed an increase in open arms exploration (percentage of entries and time spent) during a 10-min session. This effect was observed when the oil was administered before (24 h before elevated plus-maze), or after the water stress. However, this effect was not observed in non-stressed mice. The data suggest an anxiolytic-like effect that is sustained for 24 h. However, no control for motor activity was employed.

### 2.26. Spiranthera odoratissima A. St. Hil.

*Spiranthera odoratissima* A. St. Hil. essential oil acutely administered to male mice (p.o.) showed an anxiolytic-like profile: in the elevated plus-maze (increase % entries and time spent in the open arms), holeboard (increase head dipping), light-dark (increased transitions between compartments and time spent in the light side), and the open-field tests (increased center exploration), without effects on motor activity [68]. It was observed that the anxiolytic-like effect of *S. odoratissima* is partially blocked by NAN-190 (5-HT1A antagonist) but not by flumazenil, suggesting that it is related to 5-HT1A receptors but not to the benzodiazepine binding site.

### 2.27. Stachys tibetica

Kumar and collaborators [69] studied the putative anxiolytic-like effect of oral administration of *S. tibetica* essential oil in rats (male and female). Acute and repeated (3 and 7 days) *S. tibetica* essential oil administration induced an increase in open arm exploration (% time and entries), and a decrease in protected head dipping in the elevated plus-maze test. Acute treatment increased social interaction in familiar and unfamiliar conditions, and decreased aggressive behavior. Acute *S. tibetica* essential oil also exerted an anxiolytic like effect in the holeboard (decreased latency to first head dip, and increases in number and duration of dips), and in the light/dark tests (increased time spent in the light side and in the number of transitions). These effects were seen at doses that did not decrease activity in these models (e.g., no decrease of square crossing in the holeboard test) except a decrease in the number of closed arm entries in the elevated plus-maze (with 7-days of treatment). On the whole, these results suggest an anxiolytic-like effect.

### 2.28. Thujopsis dolabrata

Matsuura and collaborators [70] used anxiety induced by restraint stress to evaluate the effect of *Thujopsis dolabrata* (hiba) essential oil inhalation. Immobilization of the rats induced a decrease in the number of entries in the open arms of the elevated plus-maze (10 min sessions). Hiba essential oil reversed the anxiogenic effect of stress at a dose that did not decrease the total distance moved. These results suggest an anxiolytic-like effect for *Thujopsis dolabrata* essential oil (although only a 10-min session of elevated plus-maze was employed).

### 2.29. Constituents from Essential Oils with Anxiolytic-Like Activity

#### 2.29.1. 1,4-Cineole

1,4-Cineole is a minor component of the essential oils of certain aromatic plants (e.g., *Salvia* spp.). Considering that other monoterpenes exhibit anxiolytic-like effect, Gomes and collaborators [71] evaluated the effect of 1,4-cineole in animal models of anxiety. They observed that 1,4-cineole (oral), and diazepam increased male mice exploration of the open arms (% entries and % time spent) in the elevated plus-maze, and increased head dipping in the holeboard test. These effects of 1,4-cineole were seen at a dose that did not affect motor activity in the open field or time of permanency in rota rod tests. Flumazenil pre-treatment blocked the effect of diazepam but not the anxiolytic-like effect of 1,4-cineole, indicating that its anxiolytic-like effect is independent from the benzodiazepine site.

#### 2.29.2. α-Asarone

Lee and collaborators [72] evaluated the effect of α-asarone, (a major component of *Acorus gramineus*), in corticosterone-induced anxiety in the elevated plus-maze and holeboard tests. Male rats treated with repeated corticosterone administration (21 days, subcutaneously) displayed a decrease in the percentage of time spent and the number of entries in the open arms of the elevated plus-maze test. Moreover, corticosterone administration decreased the head dipping time in the holeboard test. Previous treatment with α-asarone dose-dependently reversed the anxiogenic-like effect of corticosterone. These effects were seen at a dose that did not change motor activity in the elevated plus-maze test (the number of closed arm entries). However, the motor effects in the open field test were mixed, since no effect was observed in the total distance travelled (in cm), but a decrease in line crossing was found after corticosterone administration (which is reversed by α-asarone). The same pattern was seen with diazepam, the positive control used. This anxiolytic-like effect of α-asarone is associated with reversion of CRH levels, and decreases in BDNF (and its receptor TrkB) mRNA induced by corticosterone treatment.

#### 2.29.3. α-Pinene

α-Pinene is an important constituent of some essential oils with anxiolytic-like profiles (e.g., *Ducrosis anethifolia*, *Chamaecyparis obtusa*), and thus, it is evaluated alone in animal models of anxiety. Satou and collaborators [73] observed that male mice treated with α-pinene inhaled for 90 min/day (for 1, 3, or 5 days) displayed increased open arm exploration (time spent and % entries) in the elevated plus maze. There was no change in this effect of α-pinene over 5 days of treatment. Although these results suggest an anxiolytic-like effect, no measurement of motor activity was taken. The effect of α-pinene may be related to potentiation of GABA inhibition [103].

#### 2.29.4. β-Caryophyllene

β-Caryophyllene is a major component of *S. odoratissima* essential oil [68] and it is also present in propolis essential oil [62]. β-Caryophyllene has presented anxiolytic-like effects, increasing open arm exploration (increase % entries and % time spent) in the elevated plus maze, and exploration of the light side of the Light/Dark test (increase time spent in light side, and number of transitions). The dosage reported, did not change motor activity in the elevated plus-maze (number of closed arms entries), or in the holeboard test (number of line crossed). However, differently from the essential oil, the anxiolytic-like effect of β-caryophyllene was not blocked by 5-HT1A antagonist, which indicates that another component may also contribute to the anxiolytic-like effect of *Spiranthera odoratissima* A. St. Hil. [68]. Considering that β-caryophyllene is an agonist of type 2 cannabinoid receptors (CB_2_), Bahi and collaborators [74] evaluated the role of CB_2_ receptors in the anxiolytic-like effect of β-caryophyllene. They observed an anxiolytic-like effect for caryophyllene in male mice in the elevated plus-maze test (increase % open arm time and entries), in the marble-burying test (decreased number of marbles buried), and in the open-field test (increase time spent in the center), yet without any effect on motor activity (number of closed arm entries in elevated plus-maze or the number of line crossings in the open-field test). These effects were reversed by treatment with AM630, an antagonist of CB_2_ receptors, indicating that CB_2_ receptor activation plays an important role in the anxiolytic-like effect of β-caryophyllene [74].

#### 2.29.5. Citral and Myrcene

As cited above, citral and myrcene, two constituents of *Lippia alba*, were studied in the elevated plus maze and no effect was observed [75].

#### 2.29.6. Carvacrol

Carvacrol is a monoterpene phenol found in the essential oils of oregano and thyme [76]. Acute oral administration of carvacrol in male mice exerted an anxiolytic-like effect in the elevated plus maze (increased % time spent and % entries in the open arms) without impairment on motor activity in the open-field test, or motor coordination in the rota rod test [76]. The anxiolytic-like effect of carvacrol is prevented by flumazenil pre-treatment, suggesting benzodiazepine site mediation. 

#### 2.29.7. Carvacryl Acetate

Pires and collaborators [77] evaluated the effect of carvacryl acetate, which is derived from carvacrol (a component of *Origanum vulgare*). Carvacrol has shown anxiolytic-like effect in animal models [76]. Male mice treated with carvacryl acetate (i.p.) showed an anxiolytic-like effect in the elevated plus maze (increase time spent and number of entries in the open arms), the light/dark test (increased time spent and number of entries in the light side), and the marble burying test (decrease in the number of marbles buried). The doses of carvacryl acetate given did not reduce motor activity in the open field test. Buspirone and diazepam were used as positive controls. The anxiolytic-like effects of cavacryl acetate were blocked by pre-treatment with flumazenil (benzodiazepine site antagonist), but not with WAY100635 (5-HT1A antagonist), indicating a GABA-A/benzodiazepine mediation.

#### 2.29.8. (*R*)-(−)-Carvone

Male rats were treated with (*R*)-(−)-carvone for 14 days (i.p.), and then submitted to the elevated-T maze [57] test. Carvone reduced avoidance latency without any effect on escape time, a profile similar to the reference drug diazepam, and to *Lippia alba* essential oil. No treatment effects were seen in motor activity in the open-field test. The results suggest an anxiolytic-like effect for carvone.

#### 2.29.9. Citronellol and 2-Phenethyl Alcohol

Citronellol and 2-phenethyl alcohol are important constituents of rose oil and thus their activity in animal models were evaluated [64]. Both compounds exhibit an anxiolytic-like effect in male mice as tested in the Geller conflict test (increasing punished behavior at a dose that did not change unpunished behavior), and in the Vogel test (increased punished licking). Citronellol exerted an inverted U curve in the Vogel test. These results suggest that citronellol and 2-phenethyl alcohol are the active constituents of rose oil [63]. Citronellol may act by increasing GABA’s inhibitory effect [103].

#### 2.29.10. (*E*)-Methyl Isoeugenol

(*E*)-Methyl isoeugenol is an important constituent of *Pimenta pseudocaryophyllus* essential oil. Male mice treated orally with (*E*)-methyl isoeugenol showed anxiolytic-like effect in the elevated plus maze (increase time spent and % entries in the open arms), in the light/dark test (increased number of transitions and time spent in light side), and in the open-field test (increased center crossings) without effects on motor activity in the open-field or wire tests [78]. Yet, (*E*)-methyl isoeugenol did not protect mice against convulsions induced by pentylenetetrazole (a GABA functional antagonist); and its anxiolytic-like effect was blocked by WAY100635 (a 5-HT1A antagonist). Thus, 5-HT neurotransmission has an important role for the anxiolytic-like effect of (*E*)-methyl isoeugenol.

#### 2.29.11. Nerol

Nerol is a monoterpene found in *Lippia* spp. and *Melissa officinalis* L. Marques and collaborators [79] evaluated the acute effect of nerol (i.p.) in male mice in the elevated plus-maze, the light-dark test, the rota-rod and the open field tests. Nerol exerted an anxiolytic-like effect in the elevated plus-maze (number of entries and time spent in the open arms), and the light-dark test (time spent in the light side). Although Nerol did not impair rota-rod performance, it did reduce motor activity, grooming and rearing in the open-field test. This later result suggests a motor impairment or sedative effect which could influence the behavior of mice in the anxiety models.

#### 2.29.12. Isopropyl *N*-Methylanthranilate and Methyl *N*-Methylanthranilate

These substances were isolated from essential oil of *Choisya ternate* and were tested in male mice submitted to the light/dark test [80]. These volatile alkaloids (administered intraperitoneally) increased the time spent in the lighter side without affecting the number of crossings. Also, these alkaloids did not affect the number of square crossings in the open field test. The results suggest anxiolytic-like effect for both isopropyl *N*-methylanthranilate and methyl *N*-methylanthranilate.

#### 2.29.13. Isopulegol

Systemic administration of isopulegol (i.p.) in male mice increased the number of entries and the time spent in the open arms of the elevated plus maze, although it also decreased the number of closed arm entries [81], which suggests motor impairment. Isopulegol also increased the number of head dips in the holeboard test. In the open field test, isopulegol did not change the number of crossings. An anxiolytic-like effect is suggested, although the putative motor effect might have influenced the results.

#### 2.29.14. (*R*)-(+)-Limonene and (+)-Limonene Epoxide

Limonene is an important constituent of some essential oils (e.g., *Citrus aurantium, Citrus junos, Citrus sinensis*, and *Lippia alba*) which has shown anxiolytic-like effect in animal models [24,25,26,27,56,57], and clinical studies [97,104]. Lima and collaborators [82] observed that (*R*)-(+)-limonene inhalation exerts an anxiolytic-like effect in male mice tested in the elevated plus-maze (increases in time spent, and the number of open arm entries), at a dose which did not interfere with motor activity (number of entries in the closed arms). Flumazenil pre-treatment did not block the anxiolytic-like effect of limonene, indicating that the benzodiazepine receptor site did not mediate this effect. Similar anxiolytic-like effect of (+)-limonene was also found by Satou and collaborators [31] who observed an increase in open arm exploration (increase in time spent and % entries) in the elevated plus-maze, and an increase in the time spent in the light side of the light/dark apparatus, without any effect on motor activity in the open-field test.

On the other hand, Vale and collaborators [75], and Costa and collaborators [27] did not observe anxiolytic-like effect for limonene in the elevated plus-maze. In both studies, diazepam, used as the positive control, increased open arm exploration, which confirms the sensitivity of the procedure.

Male mice treated orally (1 and 14 days) with (+)-limonene epoxide reduced the number of marbles buried in the marble-burying test [83]. No difference between acute and repeated treatments was seen; suggesting that tolerance did not occur for the anxiolytic-like effect of (+)-limonene epoxide. However, no measurement of motor activity was done.

#### 2.29.15. Linalool and Linalool Oxide

Linalool is a constituent of certain essential oils such as *Lavandula angustifolia*, *Piper guineense*, and *Cymbopogon citratus,* all which have shown anxiolytic-like effect. Male mice treated with linalool (inhalation for 60 min) showed increased time spent in the light side, in the light/dark test (3% linalool), and increased social interaction (1% linalool), without changes in motor activity. However, linalool also impaired step down inhibitory avoidance, indicating an amnesic effect. These effects were similar to diazepam, the positive control [84]. In this line, linalool oxide (inhaled) increased the number of entries and time spent in the open arms of the elevated plus-maze, and increased the number of crossings, and the time spent in the light side of the light/dark test [85]. However, Cline and collaborators [105] did not observe increases in open arms exploration in the elevated plus-maze after linalool administration (at 125 mg/kg, i.p.), although midazolam increased open arms exploration.

It was shown that linalool interacts with GABA-A receptors and potentiates GABA transmission [106], although it was also found that linalool did not bind to the [^3^H]muscimol (GABA-A) site [107]. Linalool also reduced [^3^H]MK801 (NMDA glutamate) cortex binding [107]. More recently, Schuwald and collaborators [55] showed that linalool prevented voltage operated calcium channel activation, similar (but not identical) to the effect of pregabalin.

#### 2.29.16. Myrtenol

Myrtenol is a compound originating from essential oil of *Myrtus communis*, a plant used in Brazil to treat “nervous conditions” [86]. Moreira and collaborators [86] evaluated the effect of (−)-myrtenol in the elevated plus-maze and light/dark test. Rats treated with myrtenol (acute, i.p.) showed increases in open arm exploration (number of entries and time spent) in the elevated plus-maze, and time spent in the light side of the light/dark apparatus. Flumazenil was able to block the effects of myrtenol. The effects of myrtenol were found at a dose that did not change motor activity in the open field, or in the rota rod tests. Interestingly, the lower dose tested was the most effective dose, which also reduced the number of closed arm entries in the elevated plus-maze. Thus, in general, (−)-myrtenol exhibited an anxiolytic-like effect, which is probably mediated by benzodiazepine-like action.

#### 2.29.17. Phytol

Phytol administration (25–75 mg/kg, i.p.) in mice (gender not specified) increased open arm exploration (number of entries and time spent) in the elevated plus-maze, and increased social interaction, while decreasing the numbers of marbles buried [87]. These effects were seen at doses that did not change the number of open field crossings, suggesting an anxiolytic-like effect. Moreover, the effect for the elevated plus-maze, social interaction, and marble burying were blocked by pre-treatment with flumazenil, indicating a benzodiazepine-like action.

#### 2.29.18. Pulegone

Pulegone, a monoterpenic compound, is found in the essential oils of *Mentha piperita* and *Mentha pulegum* L. Silveira and collaborators [88] observed that acute pulegone (i.p.) increased the percentage of time spent in the open arms for male mice submitted to the elevated plus-maze. The effect was not blocked by flumazenil pre-treatment, indicating a non-benzodiazepine meditation mechanism. However, the increase in open arm exploration was seen at a dose that also increased ambulation in the open-field. Unfortunately, the pulegone molecule is hepatotoxic [88], which precludes its use.

#### 2.29.19. Thymol

Thymol, a main component of certain aromatic plant essential oils, from s (e.g., *Origanum vulgare* and *Ocimum gratissimum* L.), was tested in female quail submitted to mechanical restraint stress [89]. Thymol was administered as a feed supplement for 2 and 15 days. Mechanical stress (wing restraint) induced immobility and consequent struggle behavior. Thymol administration reduced struggle latency and increased struggle bouts, which would be indicative of fear reduction. No effect on motor activity in an open field test was seen. Thus, the results suggest that thymol supplementation exerts an anxiolytic-like effect [89].

#### 2.29.20. Vanillin

Vanillin, a component of *Vanilla planifolia* (the vanilla bean), showed anxiolytic-like effect in male rats tested in the elevated plus-maze and light-dark tests [90]. Acute and repeated administration of vanillin (orally) increased the percentage of entries and time spent in the open arms of the elevated plus-maze and increased the number of entries, time spent, and number of rears in the bright side of light-dark test. This effect was similar to that observed with diazepam, the positive control.

### 2.30. General Discussion

Reviewing the results for essential oils in animal anxiety models, clear anxiolytic-like effect was found for *Citrus aurantium*, *Cymbopogon citratus*, *Lavendula angustifolia*, and *Lippia alba*. Although *Citrus sinensis* and bergamot oil have one pre-clinical study, clinical studies showing an acute anxiolytic effect in normal volunteers [94,97] strengthen their potential for creating new anxiolytic drugs. Putative anxiolytic-like effect was seen in: *Achillea wilhemsii*, *Angelica sinensis*, *Alpinia zerumbet*, *Celastarus paniculatus*, *Citrus janus*, *Citrus latifolia*, *Citrus reticulate*, *Cymbopogon citratus*, *Copaifera reticulate*, *Ducrosia anethifolia*, *Foeniculum vulgare*, *Spiranthera odoratissima* and *Stachys tibetica* (only one study), *Litsea cubeba*, *Ocimum gratissimum*, *Piper guineense* and *Propolis* (confounded by sedative/depressant effect, motor decrease or increase), *Abies sachalinensis*,Chamaecyparis obtuse and *Dennettia tripetala* (absence of motor activity measurement). *Citrus limon and rose oil* had contradictory results. Although there was one negative result with rose oil [34], other studies of this essential oil observed anxiolytic-like effect [64,65,66].

It is very interesting that the pre-clinical studies suggested that some essential oils do not act by the GABA/benzodiazepine mechanism. This might contribute to develop a truly new and different class of anxiolytics, possibly with a better clinical profile, and avoiding benzodiazepine’s drawbacks (e.g., withdrawal syndrome or dependence). However, even drugs acting thru the GABA/benzodiazepine system could be interesting (e.g., partial or selective benzodiazepine site agonists). In this line, the studies reviewed indicated that essential oils from *Achillea wilhemsii*, *Alpinia zerumbet*, *Lavendula angustifolia*, *Citrus aurantium*, and *Spiranthera odoratissima* did not act thru the GABA/BDZ system.

The elevated plus-maze is the most frequently used animal model, similar to that observed by Tsang and Ho [6], followed by the light/dark test, and the open-field test. One important point to consider is that animal models of anxiety may not represent the same anxiety disorder [5]. Recently, there has been growing evidence that certain animal models of anxiety are related to panic disorder (e.g., escape behavior in the elevated T-maze), obsessive-compulsive disorder (e.g., marble-burying), or generalized anxiety (e.g., avoidance in the elevated T maze or elevated plus-maze). Thus, it is important to give attention to which animal anxiety model is sensitive to which essential oil, giving a better prediction of the oil’s putative clinical efficacy for a specific anxiety disorder.

Moreover, considering the probability of false positive (and false-negative) results it is also important to evaluate a potential new anxiolytic drug in at least two different animal models of anxiety, since different models are sensitive to different anxiolytic treatments [5,6,87]. There are also criticisms to the use of the open field test to measure anxiety (increased center visiting or exploration, due to a reduction in thigmotaxis, as an index of anxiety reduction), since it lacks sensitivity to some anxiolytic treatments such as repeated antidepressants [108]. Furthermore, amphetamine, while not producing anxiolytic-like effect (on the contrary, several times it induced anxiogenic effect), increases center exploration (or center preference), which is considered an indication of increased risk-taking/poor judgment behavior, an index of a mania state [109]. It is important to note that several studies reviewed included one animal model only. Another important methodological issue is the use of a dose/concentration-response design (*i.e.*, at least two different doses/concentrations), to reduce the chance of false-negative and false-positive results.

When considering these results for essential oils, positive results in several animal models (and in different species), employing a dose/concentration-response design and a lower toxicity profile, are promising for tests in a clinical setting. Lavender essential oil is a good example, since it revealed positive results in different animal models (e.g., elevated plus-maze, marble-burying, and the social interaction tests), and in different species (e.g., rats, mice, and gerbils). It may be noted that most of the studies reviewed used acute administration, and different routes of administration (e.g., inhaled), which may be not related (or convenient) for clinical use in patients with generalized anxiety disorder. In this line, for a putative anxiolytic and chronic use, repeated systemic oral administration must be evaluated. However, it must also be stressed that acute inhaled essential oil might be used in other clinical situations, and that the approach is adequate. For example, the studies of Lehrner and collaborators [104] and Kritsidima and collaborators [110] showed an important use of inhaled essential oil for anxiety reduction in dentistry.

Attention must be given to certain methodological aspects. Motor activity must be appropriately evaluated, since changes in motor activity (e.g., sedation or muscle relaxation) can interfere with behavior in animal anxiety models. For example, although some authors proposed the total number of arm entries as a measure of activity in the elevated plus-maze [111], other authors pointed out that this measure could be contaminated by anxiety and proposed that the number of closed arm entries is a better index [112]. In the light/dark test, increase latency to first transition is sometimes taken as an index of motor impairment [113]. Thus, it is recommended that an additional test be included (e.g., open field testing). We note for the anxiety indexes used, that it would be interesting to standardize the procedures [7]. For example, in the elevated plus-maze test, (the most frequently used animal model to test anxiolytic-like drugs) [5,114] sometimes the open arm exploration is presented as a percentage of open arm entries, and as a percentage of time spent in the open arms (the most frequently reported and validated indexes of anxiety in this model). Sometimes exploration is presented as a raw measure (time in seconds, or the absolute number of entries). It would also be interesting to incorporate certain ethological measures (e.g., risk assessment), which would make the model more sensitive to non GABA/BDZ drugs [114].

Another important issue is to use a validated and standardized procedure to better translate pre-clinical results to the clinical. For example, the standard procedure for the elevated plus-maze employs a 5-min session [115] while some authors used a 10-min session, which could change the nature of behavior observed, and could lead to different results. Gender is also a relevant issue, since male and female behavior can differ in the same animal model, as seen for example in the elevated plus-maze [116]. Standardization must also be applied to open-field testing, which is a very interesting and rich, yet not well explored animal model. There are many versions of the open-field (circular, square or rectangular; black, white or transparent; under dim or bright light; motor activity measured manually, by square crossed, or even automated, using video tracking or beam interruptions), that make it difficult to compare divergent results. For example, Lamprea and collaborators [117] observed in rats an increasing preference to stay in the corner given an increasing number of walls (0, 1 or 2).

As noted by Tsang and Ho [6], it is also very important to include adequate positive controls (e.g., benzodiazepine, buspirone, or pregabalin) in order to avoid false negative results. In this line, the use of a neutral odor is interesting to control olfactory stimulation, since any new odor can trigger some behaviors by itself, or occult an aversive odor (e.g., eugenol in dentistry) [118]. Some studies included this control [37,51]. Regarding positive controls, some authors claimed that the tested essential oil is more potent or efficient or induced lower side effects than the standard anxiolytic (e.g., diazepam). This is a complicated issue since only one dose of the standard drug is administered, which does not permit conclusions in these comparisons (for lack of a dose response curve).

It has been proposed that odor has an effect, activating olfactory receptors or neurons in the vomeronasal organ, and activating limbic areas, such as the amygdala [64,65,66,82,119]. However, studies with anosmic mice [52,53], or with intraperitoneal administration (oral administration may still stimulate olfactory cues), indicate that systemic absorption (essential oil reaching the central nervous system via systemic circulation), contributes to the anxiolytic-like effects of essential oils.

## 3. Methodology

Searches were performed in the scientific literature databases: Chemical Abstracts, PubMed, and Web of Science through December 2014 using the following key words: essential oils (with each individual essential oil cited in the original articles and reviews), anxiety, animal models, (or each animal model). The search was restricted to English, and to experimental studies. An additional manual reference search in all of the articles found by the electronic search was also performed. We only included essential oils having as a minimum one study suggesting anxiolytic-like effect; essential oils with exclusively negative results were not included.

## 4. Conclusions

In conclusion, reviewing the literature of essential oils in animal models of anxiety, certain essential oils exerted anxiolytic-like effect in differing animal models and species; as such the following oils reveal the stronger potential for development of new treatments for anxiety disorders. *Achillea wilhemsii*, *Angelica sinensis*, *Alpinia zerumbet*, *Celastarus paniculatus*, *Chamaecyparis obtuse*, *Citrus aurantium*, *Citrus aurantium* subsp. *bergamia*, *Citrus janus*, *Citrus latifolia*, *Citrus reticulate*, *Citrus sinensis*, *Cymbopogon citratus*, *Copaifera reticulate*, *Ducrosia anethifolia*, *Foeniculum vulgare*, *Lavendula angustifolia*, *Lippia alba*, *Spiranthera odoratissima* and *Stachys tibetica* (only one study). Some of these essential oils are particularly interesting: *Achillea wilhemsii*, *Alpinia zerumbet*, *Lavendula angustifolia*, *Citrus aurantium*, and *Spiranthera odoratissima*,since their activity is not likely to be related to the GABA/BDZ system; and *Lavendula angustifolia*, *Citrus sinensis* and *Citrus aurantium* subsp. bergamia, since they have demonstrated clinical efficacy in controlled studies. Among the 25 constituents of essential oils with reported anxiolytic activity, no chemical characteristic was found common to all, except low molecular weight. It was not possible therefore to establish structure-activity relationships for this collection of psychoactive compounds.
